# Poly[[diaqua­bis­(μ_3_-3-carboxyl­ato-4-hy­droxy­benzene­sulfonato)­tri-μ_2_-pyrazine-tetra­silver(I)] dihydrate]

**DOI:** 10.1107/S1600536811041626

**Published:** 2011-10-22

**Authors:** Ying-Ying Liu, Shen-Tang Wang, Yong-Sheng Yan

**Affiliations:** a Department of Chemistry and Chemical Engineering, Jiangsu University, Zhenjiang 212013, People’s Republic of China

## Abstract

The title coordination polymer, {[Ag_4_(C_7_H_4_O_6_S)_2_(C_4_H_4_N_2_)_3_(H_2_O)_2_]·2H_2_O}_*n*_, contains two independent Ag^I^ ions. One Ag^I^ ion is coordinated by one O atom from a 3-carboxyl­ato-4-hy­droxy­benzene­sulfonate (*L*) ligand, two N atoms from two pyrazine ligands and a water mol­ecule. The other Ag^I^ ion is coordinated by two O atoms from two *L* ligands and one N atom from a pyrazine ligand. One of the pyrazine ligands lies on an inversion center. The *L* and pyrazine ligands link the Ag^I^ ions into polymeric layers parallel to the *ac* plane. The layers are connected by inter­molecular O—H⋯O hydrogen bonds. An intra­molecular O—H⋯O hydrogen bond is also present in the *L* ligand.

## Related literature

For a related structure, see: Nie & Qu (2011 ▶).
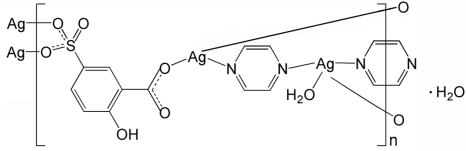


## Experimental

### 

#### Crystal data

[Ag_4_(C_7_H_4_O_6_S)_2_(C_4_H_4_N_2_)_3_(H_2_O)_2_]·2H_2_O
*M* *_r_* = 1176.14
Triclinic, 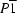

*a* = 7.646 (5) Å
*b* = 10.340 (4) Å
*c* = 11.375 (4) Å
α = 78.751 (3)°
β = 73.436 (4)°
γ = 82.882 (5)°
*V* = 843.2 (7) Å^3^
*Z* = 1
Mo *K*α radiation
μ = 2.50 mm^−1^
*T* = 293 K
0.21 × 0.15 × 0.12 mm

#### Data collection

Bruker APEX CCD diffractometer
Absorption correction: multi-scan (*SADABS*; Sheldrick, 1996 ▶) *T*_min_ = 0.622, *T*_max_ = 0.754
7253 measured reflections
3387 independent reflections
2190 reflections with *I* > 2σ(*I*)
*R*_int_ = 0.058

#### Refinement

*R*[*F* ^2^ > 2σ(*F* ^2^)] = 0.033
*wR*(*F* ^2^) = 0.073
*S* = 0.88
3387 reflections
256 parameters
5 restraints
H atoms treated by a mixture of independent and constrained refinement
Δρ_max_ = 0.70 e Å^−3^
Δρ_min_ = −1.09 e Å^−3^

### 

Data collection: *SMART* (Bruker, 2007 ▶); cell refinement: *SAINT* (Bruker, 2007 ▶); data reduction: *SAINT*; program(s) used to solve structure: *SHELXS97* (Sheldrick, 2008 ▶); program(s) used to refine structure: *SHELXL97* (Sheldrick, 2008 ▶); molecular graphics: *XP* in *SHELXTL* (Sheldrick, 2008 ▶) and *DIAMOND* (Brandenburg, 1999 ▶); software used to prepare material for publication: *SHELXTL*.

## Supplementary Material

Crystal structure: contains datablock(s) global, I. DOI: 10.1107/S1600536811041626/hy2474sup1.cif

Structure factors: contains datablock(s) I. DOI: 10.1107/S1600536811041626/hy2474Isup2.hkl

Additional supplementary materials:  crystallographic information; 3D view; checkCIF report

## Figures and Tables

**Table 1:** Selected bond lengths (Å)

Ag1—N1	2.180 (3)
Ag1—O3^i^	2.621 (3)
Ag1—O6	2.153 (3)
Ag2—N2	2.245 (3)
Ag2—N3	2.262 (3)
Ag2—O1^ii^	2.516 (4)
Ag2—O*W*2	2.576 (4)

Symmetry codes: (i) 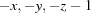; (ii) 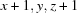.

**Table 2:** Hydrogen-bond geometry (Å, °)

*D*—H⋯*A*	*D*—H	H⋯*A*	*D*⋯*A*	*D*—H⋯*A*
O*W*1—H1*A*⋯O5	0.88 (2)	1.89 (2)	2.751 (4)	168 (6)
O*W*1—H1*B*⋯O5^iii^	0.89 (2)	2.00 (2)	2.883 (5)	173 (6)
O*W*2—H2*A*⋯O2^iii^	0.88 (2)	1.91 (3)	2.757 (5)	161 (6)
O*W*2—H2*B*⋯O*W*1^iv^	0.89 (2)	2.03 (3)	2.794 (6)	143 (2)
O4—H4*A*⋯O6	0.82	1.84	2.556 (4)	146

Symmetry codes: (iii) 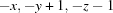; (iv) 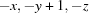.

**Fig. 1.**
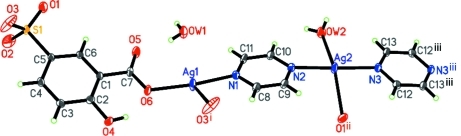
The asymmetric unit of the title compound. Displacement ellipsoids are drawn at the 30% probability level. [Symmetry codes: (i) -x, -y, -1 - z; (ii) 1 - x, -y, 1 - z; (iii) 1 - x, 1 - y, 1 - z.]

**Fig. 2.**
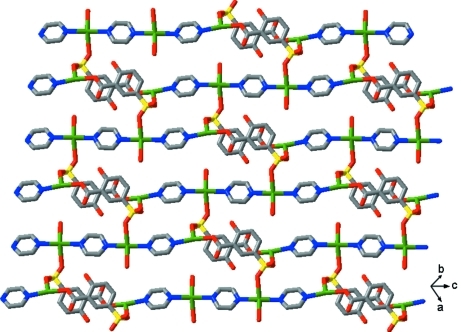
View of the two-dimensional layer in the title compound.

## supplementary crystallographic information

### Comment

As part of an investigation of the applications of transition metal complexes, there is a need to prepare further examples of these compounds. In this paper, the structure of the title compound is described.

As shown in Fig. 1, there exist two crystallographically independent Ag^I^ ions. Ag1 atom is three-coordinated (Table 1), having an approximate T-shaped geometry composed of one sulfonate O atom, one carboxylate O atom from two 3-carboxylate-4-hydroxybenzenesulfonate (*L*) ligands and one N atom from a pyrazine ligand. Ag2 atom is coordinated by one sulfonate O atom of an *L* ligand, two N atoms from two pyrazine ligands and one water molecule (Nie & Qu, 2011). The Ag^I^ ions are bridged by the *L* and pyrazine ligands, forming a two-dimensional polymeric layer (Fig. 2). The layers are connected by intermolecular O—H···O hydrogen bonds (Table 2). An intramolecular O—H···O hydrogen bond is present in the *L* ligand.

### Experimental

To a mixture of 5-sulfosalicylic acid (0.109 g, 0.5 mmol) and NaOH (0.040 g, 1.0 mmol) in water (5 ml) was added AgNO_3_ (0.170 g, 1.0 mmol), giving a clear solution. Then ethanol (15 ml) was added to the solution, and white precipitate appeared. The precipitate was collected and dissolved in water. To the solution was added pyrazine (0.081 g, 1 mmol) in methanol (5 ml) and white precipitate formed. The precipitate was dissolved by dropwise addition of acetonitrile. Colorless crystals were obtained from the filtrate after standing in a dark room for several days (yield: 0.150 g, 51%).

### Refinement

H atoms bound to C atoms and hydroxyl O atom were positioned geometrically and refined using a riding model, with C—H = 0.93 and O—H = 0.82 Å and with *U*_iso_(H) = 1.2(1.5 for hydroxyl)*U*_eq_(C, O). Water H atoms were located from a difference Fourier map and refined with a retraint of O—H = 0.88 Å and with *U*_iso_(H) = 1.5*U*_eq_(O).

### Crystal data

**Table:**

[Ag_4_(C_7_H_4_O_6_S)_2_(C_4_H_4_N_2_)_3_(H_2_O)_2_]·2H_2_O	*Z* = 1
*M**_r_* = 1176.14	*F*(000) = 574
Triclinic, *P*1	*D*_x_ = 2.316 Mg m^−^^3^
Hall symbol: -P 1	Mo *K*α radiation, λ = 0.71073 Å
*a* = 7.646 (5) Å	Cell parameters from 2192 reflections
*b* = 10.340 (4) Å	θ = 3.2–27.5°
*c* = 11.375 (4) Å	µ = 2.50 mm^−^^1^
α = 78.751 (3)°	*T* = 293 K
β = 73.436 (4)°	Block, colorless
γ = 82.882 (5)°	0.21 × 0.15 × 0.12 mm
*V* = 843.2 (7) Å^3^	

### Data collection

**Table:**

Bruker APEX CCD diffractometer	3387 independent reflections
Radiation source: fine-focus sealed tube	2190 reflections with *I* > 2σ(*I*)
graphite	*R*_int_ = 0.058
φ and ω scans	θ_max_ = 27.5°, θ_min_ = 3.2°
Absorption correction: multi-scan (*SADABS*; Sheldrick, 1996)	*h* = −9→9
*T*_min_ = 0.622, *T*_max_ = 0.754	*k* = −12→13
7253 measured reflections	*l* = −12→14

### Refinement

**Table:**

Refinement on *F*^2^	Primary atom site location: structure-invariant direct methods
Least-squares matrix: full	Secondary atom site location: difference Fourier map
*R*[*F*^2^ > 2σ(*F*^2^)] = 0.033	Hydrogen site location: inferred from neighbouring sites
*wR*(*F*^2^) = 0.073	H atoms treated by a mixture of independent and constrained refinement
*S* = 0.88	*w* = 1/[σ^2^(*F*_o_^2^) + (0.0213*P*)^2^] where *P* = (*F*_o_^2^ + 2*F*_c_^2^)/3
3387 reflections	(Δ/σ)_max_ < 0.001
256 parameters	Δρ_max_ = 0.70 e Å^−^^3^
5 restraints	Δρ_min_ = −1.09 e Å^−^^3^

### Fractional atomic coordinates and isotropic or equivalent isotropic displacement parameters (Å^2^)

**Table:**

	*x*	*y*	*z*	*U*_iso_*/*U*_eq_	
Ag1	0.29090 (6)	0.17665 (4)	−0.29686 (4)	0.03972 (13)	
Ag2	0.39877 (5)	0.41619 (4)	0.22945 (4)	0.03870 (13)	
C1	0.1323 (5)	0.1138 (4)	−0.6235 (4)	0.0216 (9)	
C2	0.2275 (6)	−0.0059 (4)	−0.6494 (4)	0.0253 (10)	
C3	0.1985 (6)	−0.0669 (4)	−0.7399 (4)	0.0288 (10)	
H3	0.2663	−0.1448	−0.7589	0.035*	
C4	0.0686 (6)	−0.0115 (4)	−0.8019 (4)	0.0263 (10)	
H4	0.0493	−0.0518	−0.8630	0.032*	
C5	−0.0332 (5)	0.1047 (4)	−0.7726 (4)	0.0219 (9)	
C6	−0.0001 (5)	0.1680 (4)	−0.6871 (4)	0.0214 (9)	
H6	−0.0656	0.2474	−0.6709	0.026*	
C7	0.1616 (6)	0.1842 (5)	−0.5288 (4)	0.0266 (10)	
C8	0.3541 (6)	0.1792 (5)	−0.0457 (4)	0.0311 (11)	
H8	0.3830	0.0903	−0.0517	0.037*	
C9	0.3716 (6)	0.2241 (4)	0.0570 (4)	0.0299 (11)	
H9	0.4142	0.1646	0.1171	0.036*	
C10	0.2678 (6)	0.4298 (5)	−0.0175 (4)	0.0317 (11)	
H10	0.2345	0.5181	−0.0100	0.038*	
C11	0.2525 (6)	0.3863 (5)	−0.1201 (5)	0.0322 (11)	
H11	0.2101	0.4459	−0.1802	0.039*	
C12	0.5470 (6)	0.3813 (5)	0.4676 (4)	0.0288 (10)	
H12	0.5817	0.2972	0.4474	0.035*	
C13	0.4107 (6)	0.5879 (5)	0.4325 (4)	0.0294 (11)	
H13	0.3477	0.6518	0.3871	0.035*	
N1	0.2969 (5)	0.2599 (4)	−0.1363 (3)	0.0280 (9)	
N2	0.3295 (5)	0.3496 (4)	0.0728 (4)	0.0281 (9)	
N3	0.4568 (5)	0.4690 (4)	0.3978 (3)	0.0288 (9)	
O1	−0.3073 (6)	0.2729 (4)	−0.7854 (4)	0.0730 (16)	
O2	−0.1208 (6)	0.2172 (5)	−0.9749 (4)	0.0738 (14)	
O3	−0.3110 (6)	0.0589 (4)	−0.8372 (5)	0.0805 (17)	
O4	0.3512 (4)	−0.0688 (3)	−0.5882 (3)	0.0359 (8)	
H4A	0.3588	−0.0253	−0.5370	0.054*	
O5	0.0833 (5)	0.2950 (3)	−0.5153 (3)	0.0421 (9)	
O6	0.2710 (4)	0.1232 (3)	−0.4660 (3)	0.0353 (8)	
S1	−0.20943 (15)	0.16850 (10)	−0.84628 (11)	0.0261 (3)	
OW1	0.0538 (5)	0.4656 (4)	−0.3518 (4)	0.0460 (10)	
H1A	0.072 (8)	0.403 (5)	−0.397 (5)	0.069*	
H1B	0.021 (8)	0.542 (3)	−0.393 (5)	0.069*	
OW2	0.2451 (5)	0.6472 (4)	0.1729 (4)	0.0449 (9)	
H2A	0.230 (8)	0.686 (5)	0.100 (3)	0.067*	
H2B	0.128 (3)	0.648 (3)	0.218 (4)	0.067*	

### Atomic displacement parameters (Å^2^)

**Table:**

	*U*^11^	*U*^22^	*U*^33^	*U*^12^	*U*^13^	*U*^23^
Ag1	0.0586 (3)	0.0419 (3)	0.0292 (2)	−0.00358 (19)	−0.0243 (2)	−0.01205 (18)
Ag2	0.0527 (2)	0.0423 (3)	0.0330 (3)	0.00471 (18)	−0.0272 (2)	−0.01620 (19)
C1	0.026 (2)	0.019 (2)	0.022 (2)	0.0003 (17)	−0.009 (2)	−0.0059 (18)
C2	0.028 (2)	0.022 (2)	0.025 (3)	0.0019 (18)	−0.011 (2)	0.0001 (19)
C3	0.034 (2)	0.023 (2)	0.030 (3)	0.0092 (19)	−0.012 (2)	−0.010 (2)
C4	0.033 (2)	0.025 (3)	0.026 (3)	−0.0005 (19)	−0.012 (2)	−0.011 (2)
C5	0.026 (2)	0.022 (2)	0.020 (2)	0.0034 (18)	−0.010 (2)	−0.0069 (18)
C6	0.026 (2)	0.019 (2)	0.020 (2)	0.0019 (17)	−0.009 (2)	−0.0044 (18)
C7	0.033 (2)	0.029 (3)	0.021 (3)	−0.004 (2)	−0.013 (2)	−0.004 (2)
C8	0.040 (3)	0.025 (3)	0.032 (3)	0.003 (2)	−0.016 (2)	−0.007 (2)
C9	0.035 (2)	0.029 (3)	0.030 (3)	0.001 (2)	−0.017 (2)	−0.004 (2)
C10	0.041 (3)	0.028 (3)	0.032 (3)	0.006 (2)	−0.020 (2)	−0.010 (2)
C11	0.040 (3)	0.031 (3)	0.031 (3)	−0.001 (2)	−0.022 (2)	−0.002 (2)
C12	0.039 (3)	0.022 (3)	0.026 (3)	0.003 (2)	−0.011 (2)	−0.006 (2)
C13	0.039 (3)	0.026 (3)	0.026 (3)	0.003 (2)	−0.015 (2)	−0.005 (2)
N1	0.031 (2)	0.032 (2)	0.024 (2)	−0.0006 (17)	−0.0104 (18)	−0.0063 (18)
N2	0.030 (2)	0.033 (2)	0.026 (2)	−0.0027 (17)	−0.0116 (19)	−0.0080 (18)
N3	0.034 (2)	0.033 (2)	0.022 (2)	−0.0005 (17)	−0.0131 (19)	−0.0043 (18)
O1	0.076 (3)	0.092 (3)	0.077 (3)	0.056 (3)	−0.058 (3)	−0.059 (3)
O2	0.066 (3)	0.111 (4)	0.036 (3)	0.010 (3)	−0.025 (2)	0.012 (2)
O3	0.082 (3)	0.041 (3)	0.143 (5)	−0.019 (2)	−0.087 (4)	0.020 (3)
O4	0.0398 (18)	0.035 (2)	0.041 (2)	0.0159 (15)	−0.0263 (18)	−0.0129 (16)
O5	0.066 (2)	0.029 (2)	0.047 (2)	0.0100 (17)	−0.037 (2)	−0.0191 (17)
O6	0.0442 (19)	0.039 (2)	0.033 (2)	0.0043 (16)	−0.0252 (18)	−0.0135 (16)
S1	0.0345 (6)	0.0234 (6)	0.0274 (7)	0.0032 (5)	−0.0195 (5)	−0.0074 (5)
OW1	0.068 (3)	0.041 (2)	0.036 (2)	0.001 (2)	−0.026 (2)	−0.0095 (18)
OW2	0.059 (2)	0.039 (2)	0.039 (2)	0.0057 (18)	−0.020 (2)	−0.0083 (18)

### Geometric parameters (Å, °)

**Table:**

Ag1—N1	2.180 (3)	C8—H8	0.9300
Ag1—O3^i^	2.621 (3)	C9—N2	1.332 (5)
Ag1—O6	2.153 (3)	C9—H9	0.9300
Ag2—N2	2.245 (3)	C10—N2	1.346 (6)
Ag2—N3	2.262 (3)	C10—C11	1.368 (6)
Ag2—O1^ii^	2.516 (4)	C10—H10	0.9300
Ag2—OW2	2.576 (4)	C11—N1	1.343 (6)
C1—C2	1.395 (6)	C11—H11	0.9300
C1—C6	1.408 (5)	C12—N3	1.343 (5)
C1—C7	1.491 (6)	C12—C13^iii^	1.369 (6)
C2—O4	1.360 (5)	C12—H12	0.9300
C2—C3	1.388 (6)	C13—N3	1.340 (5)
C3—C4	1.382 (6)	C13—C12^iii^	1.369 (6)
C3—H3	0.9300	C13—H13	0.9300
C4—C5	1.392 (6)	O1—S1	1.415 (3)
C4—H4	0.9300	O2—S1	1.444 (5)
C5—C6	1.366 (5)	O3—S1	1.423 (4)
C5—S1	1.778 (4)	O4—H4A	0.8200
C6—H6	0.9300	OW1—H1A	0.88 (2)
C7—O5	1.241 (5)	OW1—H1B	0.89 (2)
C7—O6	1.281 (5)	OW2—H2A	0.88 (2)
C8—N1	1.335 (6)	OW2—H2B	0.89 (2)
C8—C9	1.383 (6)		
			
O6—Ag1—N1	171.57 (13)	N2—C10—C11	122.4 (4)
N2—Ag2—N3	175.29 (14)	N2—C10—H10	118.8
N2—Ag2—O1^ii^	95.52 (12)	C11—C10—H10	118.8
N3—Ag2—O1^ii^	84.24 (12)	N1—C11—C10	121.9 (4)
N2—Ag2—OW2	89.83 (12)	N1—C11—H11	119.1
N3—Ag2—OW2	92.92 (12)	C10—C11—H11	119.1
O1^ii^—Ag2—OW2	147.05 (16)	N3—C12—C13^iii^	121.9 (4)
C2—C1—C6	118.4 (3)	N3—C12—H12	119.0
C2—C1—C7	122.6 (4)	C13^iii^—C12—H12	119.0
C6—C1—C7	119.0 (4)	N3—C13—C12^iii^	122.3 (4)
O4—C2—C1	122.5 (3)	N3—C13—H13	118.8
O4—C2—C3	116.8 (4)	C12^iii^—C13—H13	118.8
C1—C2—C3	120.8 (4)	C8—N1—C11	116.0 (4)
C4—C3—C2	119.8 (4)	C8—N1—Ag1	117.5 (3)
C4—C3—H3	120.1	C11—N1—Ag1	126.5 (3)
C2—C3—H3	120.1	C9—N2—C10	115.6 (4)
C3—C4—C5	119.8 (3)	C9—N2—Ag2	118.8 (3)
C3—C4—H4	120.1	C10—N2—Ag2	125.1 (3)
C5—C4—H4	120.1	C13—N3—C12	115.8 (4)
C6—C5—C4	120.6 (3)	C13—N3—Ag2	123.5 (3)
C6—C5—S1	120.4 (3)	C12—N3—Ag2	120.7 (3)
C4—C5—S1	119.1 (3)	S1—O1—Ag2^iv^	139.7 (2)
C5—C6—C1	120.5 (4)	C2—O4—H4A	109.5
C5—C6—H6	119.7	C7—O6—Ag1	123.7 (3)
C1—C6—H6	119.7	O1—S1—O3	115.8 (3)
O5—C7—O6	124.1 (3)	O1—S1—O2	110.9 (3)
O5—C7—C1	120.2 (4)	O3—S1—O2	110.4 (3)
O6—C7—C1	115.6 (4)	O1—S1—C5	106.73 (19)
N1—C8—C9	121.9 (4)	O3—S1—C5	105.6 (2)
N1—C8—H8	119.1	O2—S1—C5	106.9 (2)
C9—C8—H8	119.1	H1A—OW1—H1B	110 (6)
N2—C9—C8	122.2 (4)	Ag2—OW2—H2A	128 (4)
N2—C9—H9	118.9	Ag2—OW2—H2B	107.5 (17)
C8—C9—H9	118.9	H2A—OW2—H2B	100 (5)
			
C6—C1—C2—O4	−176.7 (4)	C11—C10—N2—C9	−1.2 (7)
C7—C1—C2—O4	1.4 (7)	C11—C10—N2—Ag2	170.5 (4)
C6—C1—C2—C3	3.1 (7)	O1^ii^—Ag2—N2—C9	35.7 (4)
C7—C1—C2—C3	−178.8 (4)	OW2—Ag2—N2—C9	−176.9 (4)
O4—C2—C3—C4	177.1 (4)	O1^ii^—Ag2—N2—C10	−135.8 (4)
C1—C2—C3—C4	−2.7 (7)	OW2—Ag2—N2—C10	11.6 (4)
C2—C3—C4—C5	−0.4 (7)	C12^iii^—C13—N3—C12	0.2 (8)
C3—C4—C5—C6	3.1 (7)	C12^iii^—C13—N3—Ag2	−177.7 (3)
C3—C4—C5—S1	−176.0 (4)	C13^iii^—C12—N3—C13	−0.2 (8)
C4—C5—C6—C1	−2.6 (7)	C13^iii^—C12—N3—Ag2	177.8 (3)
S1—C5—C6—C1	176.4 (3)	O1^ii^—Ag2—N3—C13	147.0 (4)
C2—C1—C6—C5	−0.4 (7)	OW2—Ag2—N3—C13	−0.1 (4)
C7—C1—C6—C5	−178.6 (4)	O1^ii^—Ag2—N3—C12	−30.8 (4)
C2—C1—C7—O5	174.9 (5)	OW2—Ag2—N3—C12	−177.9 (4)
C6—C1—C7—O5	−7.0 (7)	O5—C7—O6—Ag1	15.5 (7)
C2—C1—C7—O6	−4.6 (7)	C1—C7—O6—Ag1	−165.0 (3)
C6—C1—C7—O6	173.5 (4)	Ag2^iv^—O1—S1—O3	−50.2 (6)
N1—C8—C9—N2	1.2 (7)	Ag2^iv^—O1—S1—O2	76.5 (5)
N2—C10—C11—N1	0.5 (7)	Ag2^iv^—O1—S1—C5	−167.4 (4)
C9—C8—N1—C11	−1.8 (7)	C6—C5—S1—O1	−8.2 (5)
C9—C8—N1—Ag1	176.7 (3)	C4—C5—S1—O1	170.9 (4)
C10—C11—N1—C8	1.0 (7)	C6—C5—S1—O3	−132.0 (4)
C10—C11—N1—Ag1	−177.4 (4)	C4—C5—S1—O3	47.1 (5)
C8—C9—N2—C10	0.3 (7)	C6—C5—S1—O2	110.5 (4)
C8—C9—N2—Ag2	−171.9 (3)	C4—C5—S1—O2	−70.4 (4)

Symmetry codes: (i) −*x*, −*y*, −*z*−1; (ii) *x*+1, *y*, *z*+1; (iii) −*x*+1, −*y*+1, −*z*+1; (iv) *x*−1, *y*, *z*−1.

### Hydrogen-bond geometry (Å, °)

**Table:**

*D*—H···*A*	*D*—H	H···*A*	*D*···*A*	*D*—H···*A*
OW1—H1A···O5	0.88 (2)	1.89 (2)	2.751 (4)	168 (6)
OW1—H1B···O5^v^	0.89 (2)	2.00 (2)	2.883 (5)	173 (6)
OW2—H2A···O2^v^	0.88 (2)	1.91 (3)	2.757 (5)	161 (6)
OW2—H2B···OW1^vi^	0.89 (2)	2.03 (3)	2.794 (6)	143 (2)
O4—H4A···O6	0.82	1.84	2.556 (4)	146

Symmetry codes: (v) −*x*, −*y*+1, −*z*−1; (vi) −*x*, −*y*+1, −*z*.
